# Visual Quality Assessment of Posterior Capsule Opacification Using Optical Quality Analysis System (OQAS)

**DOI:** 10.1155/2017/9852195

**Published:** 2017-10-03

**Authors:** Hui Zhang, Jing Wang

**Affiliations:** Department of Ophthalmology, The Second People's Hospital of Jinan City, Jinan 250001, China

## Abstract

**Objective:**

To evaluate intraocular scattering in eyes with posterior capsule opacification by means of an objective scatter index (OSI) obtained from double-pass images of optical quality assessment system (OQAS TM II) and to determine the indication for laser capsulotomy when patients report visual disturbances without decreased visual acuity.

**Methods:**

In this prospective, observational, and nonconsecutive case series study, a total of 32 eyes of 29 patients are diagnosed with posterior capsule opacification after age-associated cataract were analyzed. Patient examination included age, the period after cataract surgery, logMAR best corrected visual acuity (BCVA), and OSI.

**Results:**

We found a significant decrease in the BCVA and increase in the OSI with the development of posterior capsule opacification. The decrease of BCVA was statistically correlated with the increase of OSI (*r* = 0.812, *P* < 0.01). In patients who reported visual disturbances without decreased visual acuity, OSI decreased to <1.3 and subjective symptoms were resolved in all cases although there was no significant improvement in visual acuity after laser capsulotomy.

**Conclusions:**

The results of our study showed that OSI is also a useful parameter for objectively evaluating posterior capsule opacification. OSI may help predict laser capsulotomy in patients who report visual disturbances without decreased visual acuity.

## 1. Introduction

Phacoemulsification combined with foldable intraocular lens implantation has become the main means for the treatment of cataract. However, postoperative posterior capsular opacification is still an important factor affecting the therapeutic efficacy. It has been reported the incidence of posterior capsular opacification in patients with the age of less than 65 years is as high as 37% [1]. At present, laser capsulotomy is still the most commonly used means in the treatment of posterior capsule opacification [2]. With the development of cataract microsurgery and improvement in the materials and design of intraocular lens, the incidence of posterior capsule opacification and laser capsulotomy was significantly reduced [3]. However, posterior capsule opacification is still a common complication after cataract surgery.

As laser capsulotomy is also associated with intraocular lens deviation, increased intraocular pressure, uveitis, macular edema, and even retinal detachment and other complications [2, 4], and the accurate and objective of evaluation of posterior capsule opacification have important clinical significance to guide its further laser treatment.

As a new visual quality analysis system, Optical Quality Analysis System (OQAS) makes objective and comprehensive evaluation of visual quality on double-pass technique. Currently, OQAS is widely used to make an objective evaluation on the grading of cataract and assist the prediction of the most optimal timing for surgical treatment. In this study, we conducted a prospective study to determine if OQAS can also provide the same objective guidance for the treatment of posterior capsule opacification.

## 2. Materials and Methods

### 2.1. Subjects

Patients with posterior capsular cataract were collected from October 2016 to April 2017 in the Second People's Hospital of Jinan City. All operations were performed by the same surgeon (Dr. Wang). The protocol was reviewed and approved by the Institutional Ethics Committee of The Second People's Hospital of Jinan. Each participant provided written informed consent before any study-related examination or procedure was performed, and the study adhered to the tenets of the Declaration of Helsinki. Patients with diabetes, glaucoma, ocular surface disease, high myopia, trauma, and previous eye surgery that affects visual acuity were excluded. The main observation parameters included the patient's gender, age, postoperative time of cataract, BCVA measured with a standard logMAR visual acuity, and OSI (objective scatter index) in OQAS. For patients undergoing laser capsulotomy, OSI both before and after laser capsulotomy was recorded. At the same time, we focused on the observation of the patients who reported visual disturbances without decreased visual acuity, and a questionnaire about visual disturbance was also performed by everyone before and after laser capsulotomy. OSI before and after laser capsulotomy was recorded and compared for these patients.

### 2.2. Examination

Routine examination included uncorrected visual acuity, intraocular pressure, B ultrasound, the best corrected visual acuity, slit lamp examination, and mydriasis fundus examination.

For OQAS examination, OSI is measured using a double-pass system (OQAS II Visual Quality Analysis System). Examination was conducted without dilation. The pupil diameter is set at 4 mm, and the patient's refractive error state can be corrected between −8 D and +6 D. Patients' astigmatism can be corrected by placing appropriate cylindrical lens before the eye. As the quality of the tear film may affect the light scattering, all the examinations were conducted after an eye blink.

After OQAS examination, 1% compound tropicamide was dropped to the patients' conjunctival sac to dilate the pupil. After mydriasis, pictures of the posterior capsule were taken. For patients who reported visual disturbances without decreased visual acuity, optical coherence tomography (OCT) needs to be improved to exclude early macular degeneration.

### 2.3. Statistical Analysis

All data were expressed as the mean ± standard deviation. Statistical analysis SPSS16.0 package was used for statistical analysis. Two-independent-sample nonparametric Mann–Whitney *U* test was used to compare OSI before and after laser capsulotomy. The relationship between variables was analyzed using the bivariate correlation model and the Pearson correlation coefficient. A *P* value less than 0.05 was considered statistically significant.

## 3. Results

The results of this study included a total of 32 eyes in 29 patients (male/female: 12/17), with the average age of 66.72 ± 8.51 years (50–88 years). Posterior capsule opacification occurred after an average of 1.94 ± 1.10 years (0.5–4 years) of cataract surgery.

### 3.1. The Relationship between the BCVA and OSI

**Figure 1:**
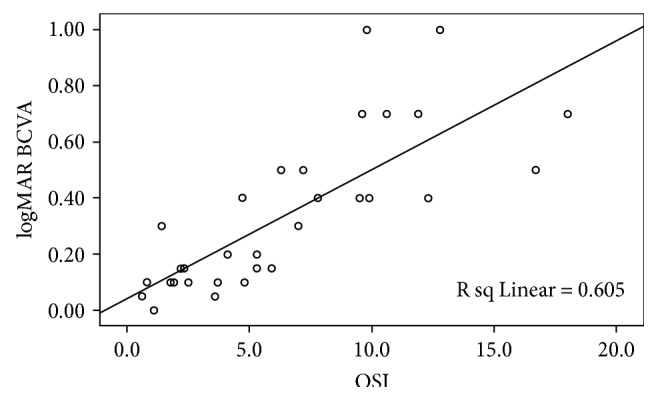
A scatter plot of the correlation between OSI and the logMAR BCVA. A positive linear correlation was found.

The BCVA of the patient was closely correlated to OSI (Pearson's correlation coefficient *r* = 0.778, *P* < 0.01). As the OSI increased, the patient's logMAR BCVA increased gradually, as shown in the scatter plot in Figure 1. The BCVA of 15 eyes was more than or equal to 0.4, with an average OSI of 10.4 ± 3.57 (4.7–18). The BCVA of 4 eyes was significantly decreased, while OSI was decreased 1 week after laser capsulotomy compared to that before laser capsulotomy (Table 1) (*Z* = −2.309, *P* = 0.029).

### 3.2. The Relationship between OSI and the Patients' Age and Postoperative Time of Cataract

There was no significant correlation between the age and OSI (Pearson's correlation coefficient *r* = 0.145, *P* = 0.437). There was also no significant correlation between the postoperative time of cataract and OSI (the Pearson correlation coefficient *r* = 0.257, *P* = 0.155).

### 3.3. OSI in Eyes with Relatively Better Visual Acuity before and after the Laser Capsulotomy

In this study, there were 13 eyes with the logMAR BCVA of 0.15 or lower than 0.15. Among them, 5 eyes had obvious symptoms of subjective visual disturbance. After OCT examination, macular disease was excluded. Subsequently, all the 5 eyes underwent laser capsulotomy. Visual acuity and OSI before and after 1 week of laser capsulotomy were shown in Table 2. Although the BCVA did not improve significantly, the subjective visual disturbance improved significantly according to the questionnaire, and the postoperative OSI improved significantly compared to the preoperative OSI (*Z* = −2.611, *P* = 0.008).

## 4. Discussion

For posterior capsule opacification evaluation, slit lamp combined with objective visual examination is still the main clinical method. However, this method of evaluation is more dependent on the subjective experience of the examiner, and thus it is more likely to make clinical mistakes. Furthermore, some posterior capsule opacifications identified by slit lamp are not associated with significant visual acuity decline. These patients have subjective blurred symptoms and require improvement in the visual function. Whether laser capsulotomy needs to be performed for these patients is still a question for the doctors. With the continuous development of digital image and computer processing, some objective quantitative evaluation of posterior capsule opacification was applied [5–7] in order to achieve more accurate and intuitive evaluation of posterior capsule opacification. However, due to the complexity of the operation and occurrence of errors, these approaches have not been widely applied. Recently, the double-pass-based objective visual quality analysis systems have been applied to objectively evaluate cataract [8–10]. Among them, OSI can quantitatively analyze the extent of cataract and has the characteristics of high accuracy, simple operation, and excellent clinical application. Our study found that it also has a good objective evaluation of posterior capsule opacification.

The presence of posterior capsule opacification reduces the patient's visual quality and is manifested as an increase in image scattering index in the double-pass system. As shown in Figure 1, with the development of posterior capsule opacification, the BCVA of the patient is declining, while the corresponding OSI is significantly increased. There is a close correlation between the decline of BCVA and the increase of OSI. However, each individual patient is different. As shown in the scatter plot, the same visual acuity can correspond to different OSI values, and the same OSI value can also correspond to different BCVA. We believe that posterior capsular opacification is unevenly distributed, and its vision and OSI values can vary differently. For patients with a central transparent gap, the BCVA can be relatively good. For those patients with 4 mm pupil diameter, scattering effect should be measured and visual function decline can be detected early. This can also explain why some patients have good BCVA, but the subjective visual disturbance is obvious.

Laser capsulotomy is a common method for the treatment of posterior capsular opacification in adults. In our study, 4 eyes with logMAR visual acuity more than 0.4 underwent laser capsulotomy and postoperative follow-up was performed. The BCVA improved, and the OSI was significantly decreased after laser capsulotomy. The other 11 eyes were not analyzed due to the lack of laser capsulotomy or laser capsulotomy performed in other hospitals. Of course, for these patients, the traditional slit lamp examination combined with reduced visual acuity can easily help physicians perform active laser treatment.

Yotsukura et al. [11] performed laser capsulotomy for 16 posterior capsule opacification patients with good BCVA, and the results showed that BCVA, contrast sensitivity, and retinal scattering were significantly improved after the operation. In this study, we performed laser capsulotomy for 5 eyes with good visual acuity and our OSI from double-pass images of optical quality assessment system also found that the visual quality was significantly improved after the operation. Although the BCVA did not have obvious improvement, patients' subjective symptoms improved significantly. As mentioned earlier, these patients maintained their BCVA due to the presence of a transparent gap in the posterior capsule. However, the patient's visual function has been affected and that can be identified by OSI, which provide foundations for laser capsulotomy. Also, our results suggested that there may have “medicolegal” advantages when considering capsulotomy in these patients, especially in medical tangle cases.

In this study, the mean age of the patients was 66.72 ± 8.51 years (50–88 years). Posterior capsule opacification occurs after an average of 1.94 ± 1.10 years of surgery. Although studies have shown that the incidence of posterior capsule opacification tends to be higher in younger patients and patients after longer time of surgery [1, 12], we found no correlation between OSI and the patient's age or between OSI and the time after cataract surgery. However, we do not exclude the possibility that this is caused by the small sample size in this study. Future studies will include a large number of subject to further verify that conclusion.

When applying OQAS for objective evaluation of cataracts, Artal et al. [10] showed that OSI > 3 can be used as a reference for cataract surgery. Due to the limited sample size, we were not able to set the OSI reference value for laser capsulotomy. However, in this study, all posterior capsule opacification patients undergoing laser capsulotomy had an OSI of being higher than 3.5. We propose that in the objective evaluation of posterior capsule opacification, the OSI > 3 for cataract surgery can be used for laser capsulotomy, which needs to be further verified in the future.

## 5. Conclusions

In conclusion, we believe that OSI from double-pass images of OQAS can be used to evaluate the posterior capsule opacification. Such evaluation is simple, concise, accurate, and reproducible and is not significantly affected by the operators or patients. Especially for patients with posterior capsule opacification and better visual acuity, OSI from OQAS can provide strong evidence for the indication of laser capsulotomy.

## Figures and Tables

**Table 1:** OSI and the BCVA in poor visual acuity eyes before and after laser capsulotomy.

	Before laser capsulotomy		After laser capsulotomy	
	logMAR BCVA	OSI	logMAR BCVA	OSI
Eye 1	1.0	9.8	0.2	0.9
Eye 2	0.7	18	0.3	4.3
Eye 3	0.5	7.2	0.2	1.6
Eye 4	0.4	12.3	0.05	0.6

**Table 2:** OSI and the BCVA in good visual acuity eyes before and after laser capsulotomy.

	Before laser capsulotomy		After laser capsulotomy	
	logMAR BCVA	OSI	logMAR BCVA	OSI
Eye 1	0.15	4.1	0.15	1.3
Eye 2	0.15	5.9	0.1	0.8
Eye 3	0.1	3.7	0.1	1.1
Eye 4	0.1	4.8	0.1	0.6
Eye 5	0.15	5.3	0.1	0.9
